# Neuroauditory Toxicity of Artemisinin Combination Therapies—Have Safety Concerns Been Addressed?

**DOI:** 10.4269/ajtmh.13-0702

**Published:** 2014-07-02

**Authors:** Virginia Ramos-Martín, Carmen González-Martínez, Ian Mackenzie, Joachim Schmutzhard, Cheryl Pace, David G. Lalloo, Dianne J. Terlouw

**Affiliations:** Department of Children's and Women's Health, Institute of Translational Medicine, University of Liverpool, Liverpool, United Kingdom; Department of Clinical Sciences, Liverpool School of Tropical Medicine, Liverpool, United Kingdom; Malawi–Liverpool Wellcome Trust Clinical Research Programme, Blantyre, Malawi; University of Liverpool, Liverpool, United Kingdom; Department of Otorhinolaryngology, Medical University Innsbruck, Innsbruck, Austria

## Abstract

Although artemisinin-based combination therapies (ACTs) are widely viewed as safe drugs with a wide therapeutic dose range, concerns about neuroauditory safety of artemisinins arose during their development. A decade ago, reviews of human data suggested a potential neuro-ototoxic effect, but the validity of these findings was questioned. With 5–10 years of programmatic use, emerging artemisinin-tolerant falciparum malaria in southeast Asia, and the first calls to consider an increased dose of artemisinins, we review neuroauditory safety data on ACTs to treat uncomplicated falciparum malaria. Fifteen studies reported a neurological or auditory assessment. The large heterogeneity of neuro-ototoxic end points and assessment methodologies and the descriptive nature of assessments hampered a formal meta-analysis and definitive conclusions, but they highlight the persistent lack of data from young children. This subgroup is potentially most vulnerable to any neuroauditory toxicity because of their development stage, increased malaria susceptibility, and repeated ACT exposure in settings lacking robust safety monitoring.

## Background

Over the past decade, artemisinin-based combination therapies (ACTs) have been deployed as first- and second-line treatments for uncomplicated malaria across malaria-endemic regions. Since 2001, this deployment has included the delivery of over 500 million treatments of artemether-lumefantrine (AL), making it one of the most widely prescribed drugs worldwide.^1^

Artemisinin derivatives are generally viewed as safe drugs with a very wide therapeutic dose range. However, a number of animal studies conducted before the wide deployment of artemisinins identified their potential ototoxic effects targeting mainly the auditory and vestibular pathways. Damage to specific brainstem nuclei was reported when administering high and parenteral doses of the lipophilic artemisinin molecules arteether and artemether.^2^–^8^ A drug treatment safety study in rats showed the drugs' toxicities during a critical neurodevelopmental stage and their potential long-term cumulative effect.^9^ This study showed that repeated treatment (up to eight cycles) with lower parenteral doses of B-arteether (5–10 mg/kg) was associated with brainstem damage after five cycles, whereas higher single doses (60–90 mg/kg) caused death without brainstem pathology.^9^ These findings suggest that repeated treatment of these oil-based artemisinin components could cause similar neurological and ototoxic damage in young children. It was argued that the implications for the use of ACTs in humans were not clear because of the use of lower doses, the use of more water-soluble compounds, and the largely oral route of administration. In programmatic settings, where malaria incidence is highest among the youngest children, individual doses of 5–10 mg/kg will occur where ACTs are dosed by age rather than weight. The possibility of cumulative ototoxicity with repeated use and the specific risk in subgroups that could be exposed to doses over 5 mg/kg have not been investigated.

Given the considerable number of trials in children 6–59 months of age and their wide-scale use in control programs, one might assume that any specific safety concerns would have been previously identified, which is not necessarily the case. Standard phase III trials generally use the drug of interest at the target dose during a single or a low number of exposures, and they are underpowered for safety end points. Furthermore, there are no standardized guidelines to evaluate ACT ototoxicity or neurotoxicity in clinical trials, both of which are more challenging to assess in young children. Without specific encouragement or recommendation of neuroauditory assessments, few research teams voluntarily opt to conduct such assessments. Pharmacovigilance conducted as part of phase IV or post-marketing safety monitoring largely depends on passive detection of adverse events within regular healthcare systems that often lack the capacity to diagnose changes in auditory function.

Over the past decades, there has been a considerable increase in the reported prevalence of hearing impairment worldwide. This increase has been attributed to better case finding, ageing populations, an increase in noise-induced hearing loss, excessive use of ototoxic drugs, and untreated otitis media.^10^ Two-thirds of the cases are from low-income countries, where preventable factors are still the leading cause (World Health Organization [WHO], unpublished data). Data on drug-induced hearing impairment again rely predominantly on relatively weak pharmacovigilance systems. Hearing impairment may not be commonly attributed to recently used drugs, resulting in underreporting. Case reports of hearing loss caused by the use of potentially ototoxic drugs are complicated further by the use of concomitant and different ototoxic drugs over time.

Techniques, such as otoacoustic emissions (OAEs), electrocochleography, and auditory brainstem responses (ABRs), have been used for detecting and monitoring ototoxicity in infants and non-responsive subjects with success.^11^,^12^ Among these techniques, ABR is viewed as the most objective and sensitive method capable of exploring the specific brainstem damage pattern formed by ACTs; the exact clinical implication of changes in the responses is, however, still not known.^13^ To assure quality ABR testing, methods are required to rule out external or middle ear disease. Tympanometry provides functional and quantitative information about the middle ear, and its use has been recommended combined with qualitative data provided by otoscopy. Nevertheless, its interpretation and reliability are equivocal in infants because of a highly compliant ear canal.^14^ Otoscopy alone, however, allows for the prompt detection of ear canal and middle ear (tympanic membrane) abnormalities before ABR performance, even by non-medically qualified staff, and therefore, it is a feasible tool in low-resource settings.^15^

In 2004, a study from Mozambique suggested that AL (Coartem, Novartis Pharma AG, Basel, Switzerland) was associated with hearing impairment in adults being treated for uncomplicated malaria.^16^ Although this reopened a global debate on ACT safety, most attention focused on the design weaknesses of the study, detracting from a valid attempt to highlight the need to exclude any safety concerns systematically. Ten years after this debate, we present an update of the published literature on available safety data of ACTs regarding neuro-ototoxicity when treating uncomplicated malaria and identify remaining knowledge gaps.

## Methods

### Search strategy.

A search of electronic databases was conducted to identify publications on the treatment of uncomplicated malaria with ACTs that included specific reports on neuroauditory safety outcomes. Exclusion criteria were no inclusion of neuroauditory or neurological safety outcomes, studies on treatment of severe malaria, pre-clinical studies, reviews, case reports, and expert opinions. There was no exclusion regarding participants, interventions, comparisons, overall outcomes, or study design.

The search was not limited by language or year, and it was carried out using the databases of EMBASE and PubMed MEDLINE. The latest search was conducted in January of 2013. The search strategy included seven different MeSH medical subject headings (MeSH) term combinations: artemisinin combination therapy and safety, artemisinins and uncomplicated malaria and safety, artemisinins and auditory safety, artemisinins and neurological safety, artemisinins and ototoxicity, artemisinins and neurological assessment, and artemisinins and hearing assessment.

### Neuroauditory and/or neurological assessment methods.

Methods currently recommended for auditory measurements in infants and children are described in Table 1. In this review, the methods to assess neurological and neuroauditory function varied from subjective reports of hearing loss by participants or caregivers and whispered voice tests to conventional and pure tone audiometry (PTA), OAE, and/or ABR. Tympanometry and otoscopy were also conducted in some studies to rule out ear canal and middle ear disease.

We included neurological and neuropsychiatric assessments when they were reported. They were either reported as general neurological assessments or specifically described as more targeted, such as fine-finger dexterity, hand coordination, audiovestibular tests (Rinne's and Weber's tests), and/or behavioral–developmental assessments adapted for children (tone and behavior—Hammersmith, Bayley). One study reported neuropsychiatric assessments based on questionnaires to caregivers and children over 3.5 years old.

### Neuro-ototoxic end points.

Neuro-ototoxic end points varied substantially across studies and among methodologies used.

#### Hearing impairment definition with audiometry.

WHO grades of hearing impairment suggest that no impairment is reported at ≤ 25 dB, slight impairment is 26–40 dB, moderate impairment is 41–60 dB, severe impairment is 61–80 dB, and profound impairment, including deafness, is ≥ 81 dB. Hearing threshold levels are taken for the better ear as the mean of the unaided pure tone threshold levels and the frequencies of 0.5, 1, 2, and 4 kHz (decibels). In addition, hearing threshold levels (decibels) defining disabling hearing impairment are established as being > 31 dB in individuals ages < 15 years and > 41 dB in individuals ages ≥ 15 years (audiometric threshold measurements according to the international standards ISO 8253-1).

#### Ototoxicity with ABR.

There are no standardized end points to assess ototoxicity with ABR, although based on animal studies, the wave III–V latency would most likely be affected by artemisinin toxicity, and any cumulative drug exposure and toxicity effect would be expected to produce a bilateral prolongation of the I–V, I–III, and III–V interpeak latencies (IPLs).^17^ Hearing failure was defined as an IPL absolute latency in milliseconds above +2.5 SD of the mean for age in this study. McCall and others^18^ looked at latencies prolonged > 2 SD from baseline. In the study by Hutagalung and others,^19^ a difference of > 0.30 milliseconds in IPLs with age-matched controls was considered clinically significant. Carrasquilla and others^20^ used a wave III latency increase of > 0.30 milliseconds at day 7 after treatment.

#### Neurological end points.

There were no specified pre-defined general neurological end points in the reviewed papers, and outcome measures consisted of description of main findings, intensity, frequency, age distribution, onset, and resolution.

### Risk of bias assessment.

A structured data collection sheet was developed to extract data from each selected study. Study design, participants, location, auditory method of assessment, neuro-ototoxic end points reported, and main results were appraised. The reported data were appraised separately by two independent assessors (V.R.M. and C.G.M.) and evaluated for the risk of bias in individuals and across studies using The Cochrane Collaboration's tool for assessing risk of bias in randomized and non-randomized studies combined with the Agency for Healthcare Research and Quality (AHRQ) 2012 recommendations (Table 2).^21^,^22^

The heterogeneity of study end points and methods prevented any meaningful meta-analyses.

## Results

**Figure 1.**
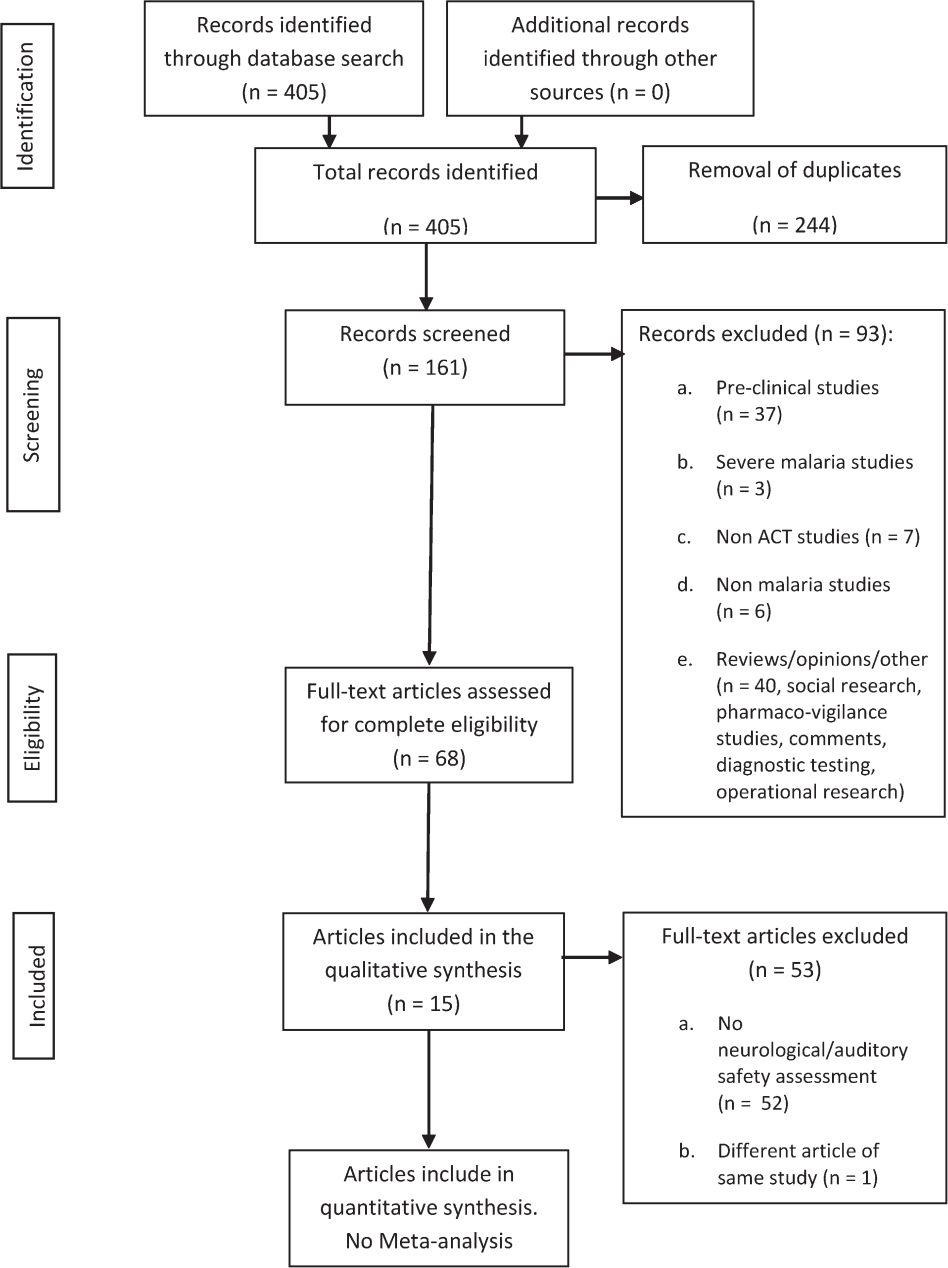
Study selection flow diagram. PRISMA 2009 Flow Diagram.

The literature search process is presented in Figure 1

**Figure 2.**
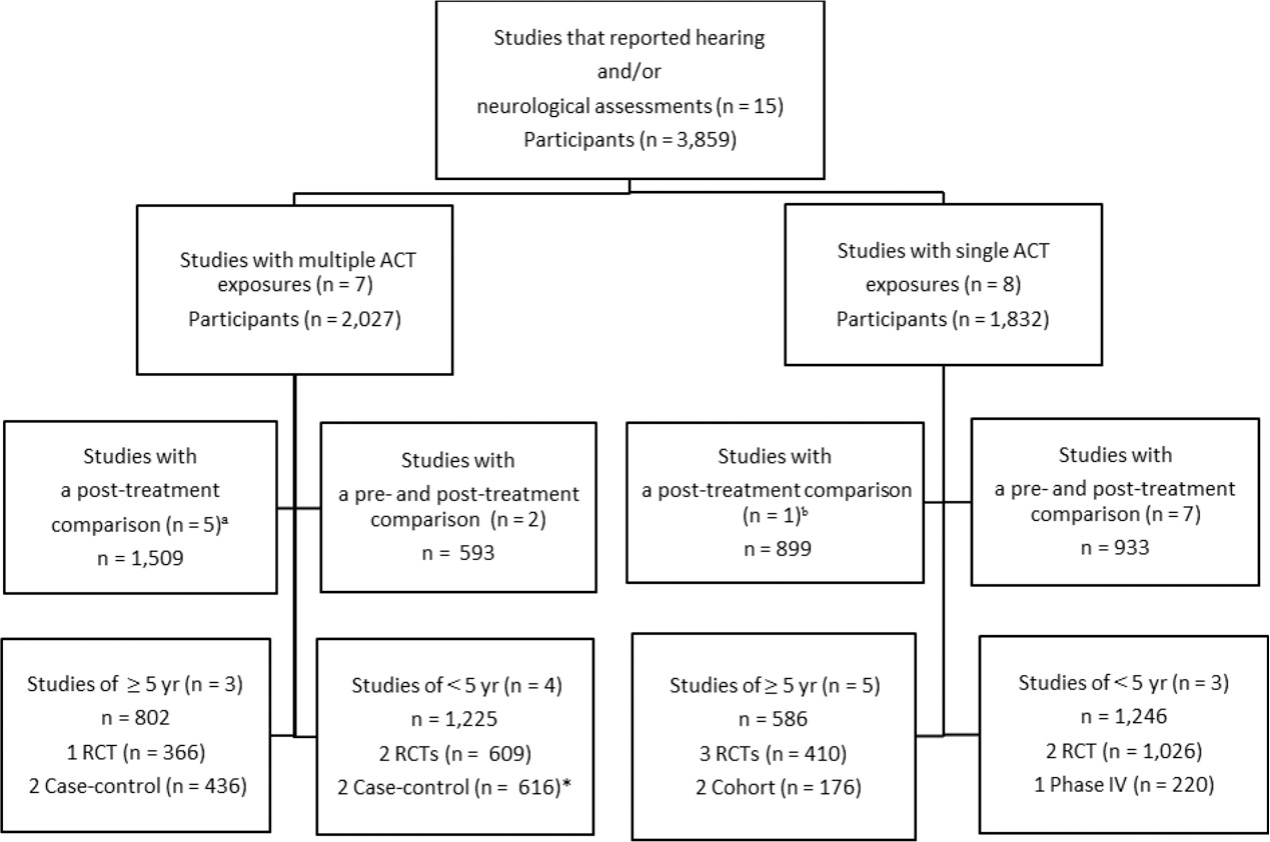
Descriptive overview of included studies. ^a^ Four retrospective studies. ^b^ Prospective study. * Only 98 cases in total were children < 5 years of age.

. Sixty-eight full-text articles were assessed for eligibility to identify specific neurological or auditory assessments that were undertaken during the safety evaluation but were not described in the abstract. Fifteen studies were eligible for inclusion in this review (eight randomized controlled trials [RCTs] and seven observational studies). Seven studies looked at multiple exposures. From these multiple exposure studies, four studies included neurological assessment (with or without auditory assessment) in children under 5 years old, and two studies were RCTs. Single exposures were investigated in eight studies: three studies included children under 5 years old, and two studies were RCTs. Seven of eight single-exposure studies conducted a pre-treatment neurological and/or auditory baseline assessment, allowing pre-to-post comparison; however, only two of the multiple exposure studies did so. The pooled studies investigated a total of 3,859 participants (including controls) (Figure 2

).

Study characteristics are summarized in Table 3, and results of individual studies are in Table 4. Of three RCTs that assessed efficacy and safety of multiple exposures to ACTs, two RCTs included children under 5 years of age,^23^,^24^ and one RCT assessed auditory safety in children ≥ 12 years old.^25^ Exposure to several courses of ACTs in these trials was not associated with an increased risk of neuroauditory adverse events. However, in one RCT that included young children conducted by Adjei and others,^23^ specific auditory assessments were only conducted in older children (> 5 years old), because the specific auditory test used relied on the cooperation and responses of the tested individual. In this study, hearing thresholds were significantly elevated in treated children compared with those thresholds in age- and sex-matched controls without malaria on days 0, 3, 7, and 28 but not after 9–12 months.^23^ Ndiaye and others^25^ performed audiometric measurements in children ≥ 12 years old during first and second malarial episodes (before treatment and on days 3 and 28) and found no significant variation on hearing thresholds at any point.

Two observational retrospective case-control studies explored the potential ototoxic effect of ACTs after multiple exposures and included children under 5 years old. In children older than 5 years (there were not enough controls under 5 years old to make comparisons), Kissinger and others^17^ and Van Vugt and others^26^ showed a prolongation in the objective ABR (longer I–III IPLs) compared with the controls without artemisinin treatment. Post-treatment ABR prolongation described by Kissinger and others^17^ was, however, only found on the right side. Neither study could correlate those changes to the cumulative dosage of artemisinins administered over periods of 2 and 3 years, respectively.^17^,^26^

Toovey and Jamieson^16^ also conducted an observational retrospective case-control study of 300 adult participants in Mozambique, where audiometry was performed on construction site workers at the start of employment and after repeated diagnosis of malaria and prescription with AL (*N* = 150). These individuals were compared with controls without drug exposure. Toovey and Jamieson^16^ found AL treatment to be associated with irreversible hearing loss. The mean time between exposure to AL and post-exposure audiogram was 163.8 days (range = 3–392 days).^16^

Among seven single-exposure studies that included auditory assessment before and after treatment, three studies reported neuroauditory abnormalities after artemisinin treatment.^20^,^27^,^28^ Gurkov and others^27^ undertook an RCT and revealed a prolongation of ABR IPLs I–III on day 28 after a single treatment with AL. This prolongation disappeared by day 90.^27^ The study also showed a significant transient cochlear hearing loss in patients treated with quinine, which has been previously reported in the literature.^27^ Carrasquilla and others^20^ also conducted an RCT assessing single exposure to AL, where 2.6% of participants showed a significant prolongation of ABR latency of wave III at day 7 after treatment (the primary outcome was wave III prolongation in ≥ 15% of participants). An additional model-based analysis found no apparent relationship between the drug exposure and the ABR changes.^20^ Finally, during a phase IV single-arm study, Frey and others^28^ found that, after a single exposure with artesunate-mefloquine (AM) in Cameroon, children aged 7 months old to 7 years old were reported to experience a transient drug-related mild to moderate neurological or neuropsychiatric impairment that resolved spontaneously. Eleven events in 8 of 213 children (5.16%) were considered to be related to the study medication^28^; the most common events were vertigo, dizziness, headache, and sleeping disorders. 28 The study could not rule out that this finding was attributable to mefloquine alone, and despite assessing neurological safety in children below 5 years old, Frey and others^28^ did not perform any auditory examination.

Lacking meta-analysis options, we summarize the current available ACT neuroauditory safety data according to the Oxford Center of Evidence Based Medicine (OCEBM) levels of evidence (Table 5). Grades B and C of evidence can be inferred uniquely from studies that tested older age groups (over 5 years old and particularly, young adults). However, in the under 5 years old age group, evidence gaps remain significant and cannot be graded.

## Discussion

This review is the first comprehensive and systematic review on specific human neuroauditory safety concerns of ACTs for the treatment of uncomplicated malaria since these concerns were raised a decade ago. Unfortunately, this review reveals a lack of collective effort to obtain the required safety data in a structured way, although ACTs have become some of the most widely used drugs in Africa over the past decade.

This review highlights the technical challenges involved in determining this specific safety concern properly, but more importantly, it questions if the scientific community has neglected the opportunity and its responsibility for ongoing targeted assessment of the safety of widely used antimalarials in real-life settings during the post-marketing period. Most of the studies aimed to assess efficacy and general safety of a single exposure. Only 15 studies looked at specific neuroauditory and/or general neurological safety outcomes. The lack of a standardized approach to drug adverse event monitoring hampered the ability to compare data collected in different studies and prevented any meaningful meta-analysis. Systematic assessments for adverse events in efficacy–safety studies generally followed the International Conference of Harmonization Good Clinical Practice guidelines. These assessments, however, did not focus on the existence of system-specific adverse events and could not rule out neuroauditory toxicity of ACTs in humans. The reviewed studies that were looking at multiple and single exposures to ACTs identified some changes in the hearing assessments performed after treatment but collectively failed to accurately and objectively investigate this finding, particularly in children under 5 years of age, most likely for very pragmatic reasons.

The investigation of hearing impairment in infants and young children can be particularly challenging, especially when trying to detect subtle and early toxicity in remote settings where trained audiologists and equipment are not available. There is a need to provide objective auditory measurements to predict the pure tone audiogram in young children that cannot report reliable behavioral responses to sound. OAEs and/or ABRs are widely used to detect sensory or conductive hearing loss in this age group (Joint Committee on Infant Hearing, unpublished data). ABR measurements are particularly well-suited in detecting and estimating a magnitude of hearing loss in young children, with click-evoked ABRs providing several advantages: they assist in determining whether auditory neuropathy exists and can be obtained in a relatively brief amount of time.^29^ However, ototoxicity tends to start at high frequencies (8–12 kHz) before speech frequencies are affected.^13^ Because conventional ABR only explores speech frequencies of 1–4 kHz, it does not detect early ototoxicity before clinical significance. The studies by McCall and others^18^ and Ndiaye and others^25^ were the only studies to explore frequencies over 8 kHz, but they involved only 12 adult patients with a single treatment of experimental human malaria and only participants ≥ 12 years old after two exposures (on day 0 before dosing, day 3, and if abnormality was detected, day 28), respectively. Neither study provided a definitive answer about ACT safety at high frequencies.^18^,^25^

The variations in study designs and auditory assessment methods used (Table 3) prevented systematic investigation of an association between neuroauditory changes and ACT use. Studies that showed impaired auditory assessments in older children, adolescents, and adults generally failed to explore the association in detail and did not examine adequately the presence of possible confounding factors. Single-exposure studies in Ethiopia and on the Thailand–Burma border, which performed pre-treatment hearing assessments to account for an influence of malaria on hearing impairment, were reassuring in their conclusions but lacked evidence. In both studies, similar improvements on hearing were detected 7 days after treatment that were correlated to a learning effect (with the use of behavioral audiometric assessments) or a fever resolution, but they failed to show or rule out an association and potential ototoxic effect of the drugs.^19^,^27^ However, the study that raised the safety concern on humans and opened a global safety discussion of ACTs reported an association between artemether and irreversible hearing impairment in construction workers. Nevertheless, the study could not establish if the association was caused by the drug, the malaria episode, or the prolonged occupational noise exposure of the study participants.^16^

Causes of hearing damage are multifactorial, and therefore, potential confounders need to be taken into account. A challenging one in this context is the potential disease-specific damage caused by malaria itself. Hearing loss is a recognized complication of cerebral malaria,^30^ but the evidence regarding an association between uncomplicated malaria and hearing impairment remains inconclusive. Some authors have hypothesized that the presence of malaria may contribute to hearing loss by lowering resistance to ototoxic drugs or vascular disruption in the end arteries of the cochlea.^31^,^32^ A recent study has assessed the impact of malaria on hearing in mice. ABRs were performed before the infection and at the peak of the disease (between days 5 and 11 after the infection without the administration of antimalarials). Hearing impairment was found in mice with both cerebral and uncomplicated malaria compared with a control group that was not infected with malaria.^33^ There is also real potential for confounding by ototoxic effects of the partner drug in the ACT, especially from quinoline-based drugs such as mefloquine, piperaquine, and amodiaquine. Mefloquine, in particular, is recognized as a central and peripheral neurotoxic, with several human reports documenting a range of neuropsychiatric effects as well as both reversible and irreversible hearing loss when used at prophylactic concentrations in adult patients.^34^,^35^ The peripheral ototoxicity of mefloquine follows a dose-dependent mechanism of cochlear hair cells and spiral ganglion neurons loss different from the artemisinin derivatives.^36^ However, animal models and a recent human case report have provided additional insight into the clinical significance and plausible pathophysiology of mefloquine focal brainstem, limbic, and thalamic cortical toxicity that needs to be emphasized and further elucidated when investigating the potential neurotoxicity of ACTs.^37^,^38^ Cognizant of this information, the US Food and Drug Administration announced label changes for the approved mefloquine hydrochloride in July of 2013 to specifically warn of the risk of permanent neurological effects, including vestibular symptoms and tinnitus.^39^

Neuroauditory events could be associated with the use of cumulative high-dose exposure in young children, and more emphasis needs to be given to the potential of dose- and age-dependent adverse events. Drugs are assessed at very narrow dose ranges during the drug development stages, and considerable developmental changes in early childhood affect the pharmacokinetic profile of drugs. With the current recommended weight-based dosing regimens in sub-Saharan Africa, children under 5 years of age could receive oral artemether ranging from 2 to 6 mg/kg per dose and from 8 to 24 mg/kg per course of treatment.^40^ In settings where age-based dosing regimens are used (e.g., treatment by village health workers or in health centers that do not have functional scales), children can be exposed to > 5 mg/kg per dose. These unintended high doses could have a detrimental neuroauditory impact that has not yet been investigated. Better knowledge and targeted studies to determine safety around the upper therapeutic intake dose threshold are urgently needed to support their programmatic use, including the development of evidence-based age-based regimens.

The main limitations of this review are the varying quality and descriptive nature of the neuroauditory findings reported by most studies and the lack of robust data from the most vulnerable groups. The risk of bias was unclear or likely for the majority of studies. Of the RCTs, only the study by Abdulla and others^41^ showed a low risk of bias in selective reporting of results. The others were classified as unclear risk of bias in this item, because safety end points were reported as secondary results without detail (Table 2). To summarize our findings, we present a summary table of results (Table 5) graded according to widely used medicine-based evidence levels (OCEMB) to help identify specific remaining neuroauditory safety gaps in the most vulnerable populations.^42^ This information highlights two important issues. First, there is a need for an appropriate standardized method to detect early ototoxicity and other adverse effects in young children. Second (and more generic), there is an urgent need to collect and compile study safety data in a more standardized way, similar to the data compilation and analyses of antimalarial efficacy data conducted by the Worldwide Antimalarial Resistance Network (WWARN).^43^

In conclusion, after a decade of use, there remains a lack of high-quality evidence on the neuroauditory safety of ACTs. There is no reported evidence to rule out the occurrence of any ototoxicity in those individuals who have been mentioned over and over again as the most vulnerable subgroup: young children who are treated repeatedly during a potentially vulnerable phase of brain development and may be exposed to some of the highest intake doses. Early evaluation and prevention of hearing impairment in childhood are essential, because hearing impairment can have severe adverse effects on speech, behavior, linguistic understanding, and language acquisition, contributing to global disability and mortality. Early-onset hearing loss detection programs are successful and increasingly implemented in the developing settings.^44^ More efforts should be made to improve this evaluation through not only universal infant hearing screening programs but also, monitoring of neuroauditory adverse events from potential ototoxic agents that are given repeatedly over time. With the current exploration of the use of ACTs for mass drug administration programs (conducting multiple full treatment courses per year) in an attempt to reduce transmission and the potential need to increase artemisinin dosing in the near future to slow the spread of emerging artemisinin resistance, this knowledge gap should not merely be accepted after a decade of widespread use but addressed as soon as possible.

## Figures and Tables

**Table 1:** Summary table of auditory methods of assessment in infants and children

Developmental age	Auditory test (time per test)	Type of conventional measurement	Strengths	Limitations and notes
Physiologic or electrophysiologic tests				
All ages	Otoacoustic emissions (10-minute test)	Cochlear (outer hair cells) response to presentation of a stimulus	Ear-specific results; not dependent on whether patient is asleep or awake; quick test time. It is a valuable screening method.	Infant/child must be relatively inactive during the test; not a true test of hearing, because it does not assess cortical processing of sound. It only tests the function of the cochlear amplifier. Damage of the cochlear amplifier results in hearing loss of approximately 40 dB.
Birth to 9 months	Auditory brainstem responses (15-minute test)*	Activity in auditory nerve and brainstem pathways after conventional (1–4 kHz) click stimulus or tone burst	Ear-specific results; responses not dependent on patient cooperation. Detects clinically significant effects that affect speech (1–4 kHz) and can interfere in neurodevelopment.	Infant or child must remain quiet during the test.
Behavioral tests				
9 months to 2.5 years	COR or VRA (30-minute test)	Responses to speech and frequency-specific stimuli presented through speakers	Assesses auditory perception of child	Assesses hearing of the better ear (not ear-specific); cannot rule out unilateral hearing loss. Subjective test: results depend on the cooperation of the patient.
2.5–4 years	Play audiometry (30-minute test)	Auditory thresholds in response to speech and frequency-specific stimuli presented through earphones and/or bone vibrator	Ear-specific results; assesses auditory perception of child	Attention span of the child may limit the amount of information obtained. Subjective testing: results depend on the will of the patient.
4 years to adolescence	Conventional audiometry (30-minute test)	Auditory thresholds in response to speech and frequency-specific stimuli presented through earphones and/or bone vibrator	Ear-specific results; assesses auditory perception of patient	Depends on the level of understanding and cooperation of the child. Subjective testing: results depend on the will of the patient.

Modified from Cunningham and Cox.^45^ COR = conditioned-oriented responses; VRA= visual-reinforced audiometry.

* Fifteen minutes is very short and can only apply if a click sound is measured at a few decibel levels. It does not explore frequencies over 4 kHz (conventional click). Therefore, it cannot detect early toxicity (ototoxic effects start in very high frequencies of 8–12 kHz). The lengths of IPLs, measured in milliseconds, are the least variable and most independent of subject stimuli and recording parameters compared with other measures derived from ABRs.^19^

**Table 2:** Risk of bias assessment of selected studies

	Selection bias		Performance bias: blinding of participants and personnel	Detection bias: blinding of outcome assessment	Attrition bias: incomplete outcome data	Reporting bias: selective reporting
	Random sequence generation	Allocation concealment				
RCTs						
Adjei and others, 2008^23^	+	?	?	?	?	?
Maiteki-Sebuguzi and others, 2008^24^	−	−	+	+	+	?
Ndiaye and others, 2011 25	−	−	+	+	+	?
Benjamin and others, 2012^46^	+	−	?	?	+	?
Carrasquilla and others, 2012^20^	−	−	+	+	−	?
Abdulla and others, 2008^41^	+	+	+	+	+	+
Gurkov and others, 2008^27^	−	−	NA	+	?	?
Ambler and others, 2009^47^	+	+	NA	+	?	?
NRTs (AHRQ)						
Kissinger and others, 2000^17^	−	NA	−	?	?	+
Van Vugt and others, 2000^26^	?	NA	?	?	−	+
Toovey and Jamieson, 2004^16^	−	NA	−	?	?	+
Hutagalung and others, 2006^19^	−	NA	−	?	?	+
McCall and others, 2006^18^	+	NA	?	?	?	+
Carrara and others, 2008^48^	+	NA	?	?	?	+
Frey and others, 2010^28^	?	NA	+	?	?	?

The Cochrane Collaboration's tool for assessing risk of bias in RCTs (adapted from Higgins and others^21^) and AHRQ recommendations (2012) for non-randomized trials (NRTs).^22^ NA = not applicable; + = low risk of bias; ? = unclear risk of bias; – = high risk of bias.

**Table 3:** Summary table of main data extracted

Study	Study design	Participants and location	Neuroauditory methods	Neuro-ototoxic end points
Multiple exposures (seven studies; including children < 5 years)				
Kissinger and others, 2000^17^	Case-control. Cases (*N* = 350): Ar/As (*N* = 242), MQ (*N* = 10), ArMQ (*N* = 98). Controls match age/sex (*N* = 108). ME range in the group (1–21), Retrosp	1–65 years; 96 cases < 5 years (compared with published normative data, because only 2 controls were < 5 years); Vietnam	Neurological examination, otoscopy, audiometry, ABR (80 dB). Patients exposed ≤ 2 years ago. Time since last Rx not reported.	Abnormalities in hearing, vestibular and cerebellar function. Intensity of drug exposure (milligrams per kilogram) effect on ABR; PTA: dB threshold at ≤ 30 to > 60 dB from 500 Hz το 500Hz–8kHz 8 kHz (4 kHz); ABR: IPLs I–V, I–III, and III–V (milliseconds). In ABRs, the equipment accuracy was 0.1 milliseconds; below this variation, no significance; > 2.5 SD of normative data mean for age was considered abnormal.
Van Vugt and others, 2000^26^	Case-control. Cases (*N* = 79). Oral Ar or As ≥ 2 in last 3 years. Controls match age/sex (*N* = 79). ME range per case (2–9), Retrosp	3–53 years (< 5 years; *N* = 2); Thailand	Neurological examination, audiometry, ABRs (80 dB). Median time (range) from most recent exposure was 385 days (31–1,963 days)	Abnormalities in neurological assessment (Romberg's test, tandem test, fine-finger dexterity, eye movements, behavior); PTA: decibel hearing threshold (0.5–8 kHz); ABR: I–III, I–V, III–V IPLs (milliseconds)
Adjei and others, 2008^23^	RCT AS-AQ (*N* = 116) vs. LA (*N* = 111; 1-year follow-up). ME (?). Incidence rates of 0.37 in AS-AQ and 0.34 in LA, Prosp, Pre-Rx	6 months to 14 years; Ghana	Neurological examination; PTA only in ≥ 5 years (*N* = 72: AS-AQ = 37; LA = 35); on days 0, 3, 7, and 28 and 1 year after	Abnormalities in neurological assessment; PTA: decibel hearing thresholds (0.125–8 kHz)
Maiteki-Sebuguzi and others, 2008^24^	RCT UM repeated Rx AQ-SP (*N* = 129) vs. AS-AQ (*N* = 133), LA (*N* = 120). ME (?), Prosp	1–12 years; Uganda	Neurological examination with hearing assessment (assessment not mentioned) and fine-finger dexterity on days 0, 1, 2, 3, 7, and 14	No neuro-ototoxic end points
Multiple exposures (not including children < 5 years)				
Toovey and Jamieson, 2004^16^	Case-control in subjects working at a construction site. Cases (*N* = 150) LA. Controls match age, sex, weight, race (*N* = 150). ME (?), Retrosp	18–72 years; Mozambique	Audiometry taken at the beginning of project and nearly 2 years after. Mean time (range) between exposure to LA and post-exposure audiogram was 163.8 days (3–392 days)	Audiometry decibel hearing thresholds(0.25–8 kHz)
Hutagalung and others, 2006^19^	Case-control. Cases (*N* = 68) Rx more than one time with AL in past 5 years. Controls (*N* = 68) match age/sex. ME (?), Retrosp	7–65 years; Thailand	Tymp, PTA, ABR (80 dB). Median time (range) between exposure and audiometry testing was 33 months (20–58 months)	Tymp: MEP (decapascal; < 150 daP excluded); PTA: decibel hearing threshold (0.25–8 KHz). Mild, moderate, and severe hearing loss if ≥ 25, ≥ 30, and ≥ 35 dB, respectively, at any tested frequency; ABR: I–III, III–V, and I–V IPLs (milliseconds). Difference > 0.30 milliseconds was considered physiologically significant.
Ndiaye and others, 2011^25^	RCT, open label with ME to AS-AQ (*N* = 184) vs. LA (*N* = 182). ME (?), Prosp, Pre-Rx	9.6 months to 65 years; Senegal (no specific neurological or auditory assessment in individuals < 5 years)	Audiograms after all malaria episodes on days 0, 3, and 28 (if any abnormality were detected on day 3); audiograms only in ≥ 12 years	Difference in decibel thresholds obtained for each ear and octave range (5–16 kHz) on days 0 and 3 (day 28 if abnormalities are detected).
Single exposures (eight; including children < 5 years)				
Abdulla and others, 2008^41^	RCT, single blind, multicenter Lad (*N* = 447) vs. LA (*N* = 452). SE, Prosp	0–12 years, ≥ 5 kg; Benin, Kenya, Mali, Tanzania, Zanzibar, Mozambique	Neurological examination; reported hearing loss by children	No auditory end points
Ambler and others, 2009^47^	RCT, open label, single center, neurological safety of As-MQ (*N* = 46) or As mono (*N* = 45). Non-febrile control children (*N* = 36). SE, Prosp. Pre-Rx	3 months to 5 years; Thailand	Neurological examination (coordination + behavior), hand coordination-adapted Griffith's Scales + Movement ABC, tone and behavior—Hammersmith, Bayley	No auditory end points
Frey and others, 2010^28^	Phase IV, open-label, single-arm study assessing the neurological and neuropsychiatric safety of As-MQ (*N* = 220). SE, Pre-Rx	7 months to 7 years + 9 months (10–20 kg); Cameroon	Neurological and neuropsychiatric examinations on days 0, 7, 28, and 63 (Q to guardian, Q to child, investigator observations, and 12 clinical examinations based on Touwen paeds neurological examination)	Questionnaires and examinations covered hearing loss and acoustic acuity. Not specified end points apart from intensity of adverse events, frequency, age distribution, onset, and resolution.
Single exposures (not including children < 5 years)				
McCall and others, 2006^18^	Cohort, volunteers (*N* = 15) undergoing experimental malaria infection and Rx LA. SE, Prosp, Pre-Rx	18–23 years (malaria-naïve); The Netherlands	Tymp day 0, DP-OAE day 0; PTA (standard + high frequency) days 0, 8, 21/22; ABR (70 dB) days 0 and 21/22	Tymp: MTC (milliliters) + MEP (decapascal); OAE: DP thresholds; PTA: decibel hearing thresholds (0.25, 0.5, 1–16 kHz); ABR: I–V IPLs (milliseconds) + I, III, and V peak latencies+ V peak ABR auditory thresholds. Latencies prolonged > 2 SD from baseline and decibel threshold deteriorations ≥ 10 dB
Carrara and others, 2008^48^	Safety study (*N* = 161; *N* = 93 in final analysis) assessing auditory functions in patients UM As-MQ day 0 (pre) vs. 7. SE, Prosp, Pre-Rx	13–53 years; Thailand–Myanmar border	Otoscopy, tymp, audiometry, ABR (80 dB)	Tymp: MEP < −150 daP or flat wave or a wave peak with oscillations (were excluded); PTA: decibel hearing thresholds (0.25–8 kHz) >10 dB between days 0 and 7; ABR: wave III latency (milliseconds) was the primary end point (difference > 0.3 milliseconds between days 0 and 7)
Gurkov and others, 2008^27^	RCT, open-label LA (*N* = 30) vs. Q (*N* = 35), AP (*N* = 32). SE, Prosp, Pre-Rx	6–50 years (not exposed to artemisinin before); Ethiopia	Audiovestibular tests, PTA, TE-OAE, DP-OAE, ABR (80 dB)	Clinical data: no pre-defined clinically relevant end point; PTA: decibel hearing thresholds by air (0.125–8 kHz) and bone conduction (0.25–6 kHz). Difference in means of 5 dB between groups assumed as common SD; TE-OAE: pass vs. fail; DP-OAE: DP thresholds; ABR: I–III, III–V, and I–V IPLs (milliseconds)
Benjamin and others, 2012^46^	RCT, single-center, single-dose ART-NQ + water (grade 1, *N* = 15), single-dose ART-NQ + milk (grade 2, *N* = 17), or two daily doses + water (grade 3, *N* = 16). SE, Prosp, Pre-Rx	5–12 years; Papua New Guinea	PTA (grade 1), Whispered voice test (grades 2 and 3). Rinne's and Weber's test (grades 2 and 3); hearing tests: day 0 (4 hours) and days 1, 7, and 28. No mention of additional neurological assessments	PTA: Difference in decibel air conduction thresholds at ≥ 25–40, 41–55, 56–70 (0.25–8 kHz), and. 71–90 dB. Whispered voice test: repetition of at least three of six numbers/letters corrected
Carrasquilla and others, 2012^20^	RCT, open-label, single-center, LA (*N* = 159), AP (*N* = 53) vs. AM (*N* = 53). SE, Prosp, Pre-Rx	12–56 years; Colombia	Tymp, otoscopy, ABR, PTA days 0 (pre-Rx), 3 (1 hour after last Rx dose), 7, 28, and 42	Primary outcome: ≥ 15% patients with a day 7 wave III latency increase > 0.30 milliseconds after LA

ABC = assessment battery for children; AP = atovaquone-proguanil; Ar/As = artemisinin or artesunate; ArMQ = artemisinin + mefloquine; ART-NQ = artemisinin-naphtoquine; AS-AQ = artesunate-amodiaquine; AQ-SP = amodiaquine-sulfadoxine-pyrimethamin; DP = distorsion product; LA = lumefantrine-artemether; LAd = LA dispersible; ME = multiple exposure; MEP = middle ear pressure; MQ = mefloquine alone; MTC = maximum tympanic compliance; pre-Rx = pre-treatment assessment; Prosp = prospective; Q = quinine; Retrosp = retrospective; Rx = treatment assessment; SE = single exposure; TE = transient-evoked; Tymp = tympanometry; UM = uncomplicated malaria; ? = not reported.

**Table 4:** Summary of main study results

Study	Relevant results
Multiple exposures	
Kissinger and others, 2000^17^	Neurological examination: no abnormalities among examined subjects (81.5% of subjects). Otoscopy: 28% had pus or perforation. ABR: in ≥ 5 year olds, very small but significant difference in I–V (3.82 vs. 3.74 milliseconds, *P* = 0.007) and I–III IPLs (2.03 vs. 1.98 milliseconds, *P* = 0.0027) of right ear (longer) in cases vs. controls. In < 4 year olds (compared with normative data), only one patient had an IPL > 2.5 SD from the mean for the age (the child also had pus in the external auditory canal). Effect of total drug exposure: IPLs I–III (2.07 vs. 2.00 milliseconds, *P* = 0.014) and I–V (3.89 vs. 3.79 milliseconds, *P* = 0.014) of the right ear of cases with higher cumulative dosage (> 500 mg/kg) vs. (≤ 500 mg/kg) Ar/As cumulative dosage. However, when correcting for age, no significant differences regarding cumulative dosage in IPLs.
Van Vugt and others, 2000^26^	Neurologic examination: all normal except for hearing test in one case and two controls (PTA and ABR). Very small but significant difference between controls and cases (longer; 2.08 vs. 2.14 milliseconds; SD = 0.19) for the IPLs I–III only in the right side (*P* = 0.049). No correlation between the total dose (milligrams per kilogram) of artemisinin administered and IPLs.
Adjei and others, 2008^23^	Neurologic examination: no abnormal findings in children without previous pathologies. PTA: hearing thresholds significantly elevated in treated children on days 0, 3, 7, and 28 but not at 9–12 months. No additional details.
Maiteki-Sebuguzi and others, 2008^24^	Neurological examination: children < 5 years who received AQ-SP were at higher risk of anorexia (RR = 3.82, 95% CI = 1.59–9.17, *P* = 0.003) and weakness (RR = 5.40, 95% CI = 1.86–15.7, *P* = 0.002) than those children treated with AL. No reported results on hearing loss.
Toovey and Jamieson, 2004^16^	PTA: hearing threshold loss was significantly greater in the treatment group at all except the very lowest frequencies of 250 and 500 Hz. The mean threshold change was negative in the treatment group, ranging from −6.50 dB (95% CI = −8.19 to −4.81) to −0.07 dB (95% CI= −2.19–2.05).
Hutagalung and others, 2006^19^	High proportion of subjects with hearing loss overall related to age. PTA: no differences between the groups in MEP and the median PTA conduction thresholds. ABR: no differences in wave length or the IPLs.
Ndiaye and others, 2011^25^	Audiograms in 167 patients during the first malaria episode and 12 patients during the second episode. Hearing thresholds on days 3 and 28 showed no significant variation compared with pre-Rx on day 0 and no difference between AS-AQ and LA.
Single exposures	
Abdulla and others, 2008^41^	Neurological examination: isolated cases of somnolence, convulsion, dyskinesia, epilepsy, dizziness, and tremor reported as unrelated adverse events. No patient reported hearing loss.
Ambler and others, 2009^47^	Neurological examination: coordination, behavior, and tone not significantly changed by either treatment.
Frey and others, 2010^28^	Neurological and neuropsychiatric examinations: among 213 children, 3.8% of the children had a transient drug-related mild to moderate neurological or neuropsychiatric impairment, which resolved spontaneously. The most common neurological disorders were sleeping disorders, insomnia, nightmares, vertigo, dizziness, and headache. No report on hearing impairment.
McCall and others, 2006^18^	Tymp, OAE, PTA, ABR: no prolongations of peak latencies or I–V IPLs were seen. No statistically significant differences after the treatment (day 8 for PTA and day 21/22 for ABR + PTA) compared with before the infection.
Carrara and others, 2008^48^	Hearing loss on admission was common (57%) and associated with age. Day 7 vs. 0 showed no threshold change > 10 dB and no shift in wave III latency > 0.30 milliseconds.
Gurkov and others, 2008^27^	PTA and DP-OAE revealed transient significant cochlear hearing loss in patients treated with Q on day 7 that disappeared on day 28. ABR: the prolongation of I–III IPLs between LA and the other groups on day 28 disappeared by day 90 (only right ear). In all groups, IPLs I–V was shorter on day 0. One patient in the LA group had a potentially clinically relevant interaural difference of IPLs I–III > 10% on day 28 (disappeared by day 90).
Benjamin and others, 2012^46^	PTA: At baseline, children had normal or mild hearing loss (≥ 25–40 dB) with no subsequent changes on audiometry over time in group 1 (*P* > 0.05). In groups 2 and 3, 76% of children cooperated with the tests. Whispered test, audiovestibular tests: no abnormalities were detected by any of the tests at baseline or subsequently.
Carrasquilla and others, 2012^20^	ABR: 2.6% of patients on LA (95% CI = 0.7–6.6) exceeded 0.30 milliseconds at day 7 wave III latency, statistically significant below 15% (*P* < 0,0001). No patient receiving AM or AP revealed day 7 III IPLs increases of > 0.30 milliseconds. None of the latency increases were sustained, bilateral, or associated with significant PTA thresholds deteriorations. PTA: no notable changes were observed for any treatment group at any frequency. A model-based analysis found no apparent relationship between drug exposure and ABR change.

CI = confidence interval; RR = relative-risk.

**Table 5:** Level of evidence of ACT neuroauditory safety

Type of safety assessment	Reversibility of neuroauditory changes found assessed*	Cumulative total drug exposure assessed	Subclinical damage/early toxicity assessed†	Any neuroaudiological assessment after single-drug exposure	Any neuroaudiological assessment after multiple drug exposure	Comprehensive neuroaudiological assessment after single-drug exposure	Comprehensive neuroaudiological assessment after multiple drug exposure	Level of evidence of ACTs neuroauditory safety OCEBM recommendations‡
*n*/Total included studies	10/15 (66.6%)	2/15 (13.3%)	2/15 (13.3%)	7/15 (46.6%)	7/15 (46.6%)	4/15 (26.6%)	3/15 (20%)	
Study design	6 RCTs; 1 Retrospective-CC; 3 Prospective-C	0 RCTs; 2 Retrospective-C	1 RCT; 1 Prospective-C	4 RCTs; 3 Prospective-C	3 RCTs; 4 Retrospective-CC	2 RCTs; 2 Prospective-C	0 RCTs; 3 Retrospective-CC	
> 15 years	+	+	+	+	+	+	+	B
5–15 years	+	+	+/–	+	+	+	+	B/C
12 months to 5 years	+/–	+/–	Not tested	+	+	Not tested	+/–	Suggested safety but not proven; on hold
6–12 months	+/–	+/–	Not tested	+/–	+/–	Not tested	Not tested	Not proven; on hold
< 6 months	Not tested	Not tested	Not tested	Not tested	Not tested	Not tested	Not tested	Not proven; on hold

C = cohort; CC = case control; + = age group fully tested; +/– = age group partially tested.

* Assessments were repeated at several time points over time.

† More than 8 kHz frequencies in PTA.

‡ OCEBM Grades of recommendations: A = consistent level 1 studies; B = consistent level 2 or 3 studies or extrapolations from level 1 studies; C = level 4 studies or extrapolations from level 2 or 3 studies; D = level 5 evidence or troublingly inconsistent or inconclusive studies of any level. Levels (therapy/preventions/etiology/harm): 1 = systematic review of RCT, individual RCT, or all/none studies; 2 = systematic review of cohort studies, individual cohort studies, or outcomes research; 3 = systematic review of CC studies or individual CC studies; 4 = case series; 5 = expert opinion.^41^
